# Quality indicators in prolonged hemodialysis with regional citrate anticoagulation with the genius system: retrospective cohort of critical patients with acute kidney injury

**DOI:** 10.1186/s12882-023-03342-8

**Published:** 2023-11-30

**Authors:** Jorge Alberto Menegasso Vieira, Isabel Cristina Reinheimer, Amanda Corrêa dos Santos, Fernando Kowarick Halperin, Luiza Aguirre Susin, Lia Portella Staub, Raquel Jaqueline Eder Ribeiro, Julia Braga da Silveira, Lucas Friedrich Fontoura, Diego Candido de Souza, Karen Patrícia Nunes, Vandrea Carla de Souza, Luciano da Silva Selistre, Carlos Eduardo Poli-de-Figueiredo

**Affiliations:** 1 Department: Nephrology Service, Pontifícia Universidade Católica do Rio Grande do Sul - PUCRS, Av. Ipiranga, 6681 - Escola de Medicina - Prédio 12, Porto Alegre, Rio Grande do Sul CEP 90619-900 Brazil; 2 Academic Master’s and Doctorate Degree in Health Sciences, Universidade de Caxias do Sul (UCS), Street Francisco Getúlio Vargas, 1130, Caxias do Sul, Rio Grande do Sul 95070-560 Brazil

**Keywords:** Regional citrate anticoagulation, SLED technique, Hybrid renal replacement therapy, Sustained low-efficiency dialysis, Slow extended dialysis mode

## Abstract

**Background:**

Prolonged hemodialysis (HD) is performed from 6 to 12 h and can last up to 24 h. To prevent system clotting some studies suggest that Regional Citrate Anticoagulation (RCA) use reduces bleeding rates relative to systemic heparin. However, there may be difficulties in the patient’s clinical management and completing the prescribed HD with Genius system using RCA.

**Objective:**

To analyze safety Quality Indicators (IQs) and follow up on prolonged HD with 4% sodium citrate solution in a Genius® hybrid system.

**Methods:**

This is a retrospective cohort conducted in an intensive care unit.

**Results:**

53 random sessions of prolonged HD with 4% sodium citrate solution of critically ill patients with AKI assessed. Evaluated safety indicators were dysnatremia and metabolic alkalosis, observed in 15% and 9.4% of the sessions, respectively. Indicators of effectiveness were system clotting which occurred in 17.3%, and the minimum completion of the prescribed HD time, which was 75.5%.

**Conclusion:**

The assessment of the indicators showed that the use of RCA with a 4% sodium citrate solution in prolonged HD with the Genius system in critically ill patients with AKI can be performed in a simple, safe, and effective way.

## Key learning points

**What is already known about the subject?:**

Studies on RCA or systemic heparin usage do not demonstrate differences in the mortality of critically ill patients with AKI undergoing acute RRT. However, they suggest lower bleeding rates and greater longevity of the dialysis filter. Nonetheless, hemodynamic stability and hydroelectrolytic and acid-base disorders of patients is the controversial point.

**What this study adds?:**

Quality Indicators show that this simplified prescription and RCA in prolonged HD, using the Genius® hybrid system, in critically ill patients with AKI is safe and effective.

**What impact this may have on practice or policy?:**

The proposed prescription is easy to implement, has low demands on the care team, and enables the use of hypertonic citrate for prolonged hemodialysis using the Genius® system, even with dialysate containing calcium. Monitoring the IQs may help improve the safety and effectiveness of the care routine.

## Introduction

Acute Kidney Injury (AKI) is common in critically ill hospitalized patients and is associated with high morbidity and mortality rates [1]. Hemodialysis (HD) is essential in these patients’ treatment, and traditionally it has been offered intermittently or continuously. However, in recent years, prolonged HD has stood out as an alternative therapy that provides safety and efficacy in treating critically ill patients with AKI [2].

Prolonged HD is conventionally performed from 6 to 12 h and can reach up to 24 h [3, 4]. It can occur in a traditional or a hybrid HD system. The hybrid system has characteristics common to intermittent systems and continuous dialysis systems. Thus, it adds cardiovascular stability and effective clearance of the continuous HD to operational facilities and reduces costs, similar to intermittent HD [5].

Dialysis therapy with the Genius® 90 hybrid system has the advantages of a closed HD system with efficiency, bacteriological safety, autonomy, and ease of movement, as the equipment has a 90-liter dialysate storage tank [6]. Such characteristics favor the use of prolonged HD in a hybrid system in Intensive Care Units (ICU) [7]. However, the anticoagulation of the extracorporeal circuit remains the major controversy in this treatment [8].

During HD, intrinsic and extrinsic pathways of the coagulation cascade are activated, with a risk of thrombosis in the system. Anticoagulant use aims to maintain the viability and performance of the dialysis equipment and filter [9, 10].

Historically, the standard anticoagulation regimen has been the systemic use of unfractionated heparin. Although well established, this practice is associated with an increased risk of bleeding, especially in critically ill patients with impaired coagulation and the risk of heparin-induced thrombocytopenia [11]. Bleeding complications occur in up to 30% of critically ill patients with AKI during systemic anticoagulation in dialysis, and this increases the risk of mortality and the need for blood transfusion in these patients [12, 13].

Regional Citrate Anticoagulation (RCA) has emerged as an alternative, and its main advantage is to avoid systemic anticoagulation in critically ill patients and at risk of bleeding or both. [14]. Some evidence of RCA use has shown a lower incidence of bleeding complications and need for transfusion, better membrane biocompatibility, and prolonged dialysis filter life [15]. Experience with the use of citrate in the Genius® system is limited, particularly because the dialysate contains calcium in its composition. There are some difficulties in using RCA in prolonged HD due to the clinical condition of the patients and the high cost of the available protocols. Some intensivists are reluctant to adopt this practice due to the complexity of electrolyte management (hypocalcemia) and metabolic complications (especially alkalosis and dysnatremia). In addition, there is a risk of citrate accumulation and the need for customized RCA solutions and replacement fluids [11, 12, 14].

Few studies still address RCA in a hybrid system with dialysis time greater than 8 h [2]. In this sense, there is a demand for updating publications, given the new anticoagulation protocols used in clinical practice and the equipment and technological resources available in dialysis.

Due to the incorporation of new devices and clinical practices into health systems, it is necessary to perform the Health Technology Assessment (HTA). Studies that help in constructing suitable methods of great relevance for HTA [16]. In this context, an approach with great applicability is the research on Quality Indicators (QIs). In this regard, the Acute Disease Quality Initiative (ADQI) has declared a priority in developing these studies due to the scarcity of evidence-based IQs in prolonged HD [17].

In our university hospital, the employment of prolonged HD with a Genius® hybrid system started in 2017, especially in ICU patients. We recently started using RCA and no critical evaluation has been carried out on the clinical results of using this technology and on RCA. Thus, the present study proposes to describe the outcomes of safety and effectiveness indicators of prolonged HD in a hybrid Genius® system using a 4% sodium citrate solution in critically ill patients with AKI.

## Materials and methods

The current study is a retrospective cohort of critically ill patients with AKI who underwent prolonged HD in a hybrid Genius® 90 system (Fresenius Medical Care, Bad Homburg, Germany) with regional anticoagulation with 4% sodium citrate (Life® - Laboratório de Insumos Farmacêuticos Estéreis Ltda) from December 2019 to December 2021 at Hospital São Lucas (HSL) of the Pontifical Catholic University of Rio Grande do Sul (PUCRS). The present paper evaluated the possible association between the combined exposure factor (prolonged HD ≥ 6 h in Genius® hybrid system + 4% sodium citrate) and primary and secondary outcomes.

Preparation of the dialysate with CPHD 138/35–90 Genius DS concentrate and CPHD Genius HC 22–90 acid solution (Fresenius Medical Care) results in a final electrolyte concentration (90 L) containing sodium 138 mmol/l, potassium 2.0 mmol /l, calcium 1.25 mmol/l, magnesium 0.5 mmol/l, chloride 110 mmol/l, citrate 0.08 mmol/l, bicarbonate 32.63 mmol/l and glucose 5.5 mmol/l. The reason for the use of a Ca-containing dialysate is because a Ca-free dialysate wasn’t available.

Sodium citrate 4% (Life®) was used for anticoagulation (citrate 116 mmol/l or 408 mEq/l; sodium 408 mmol/l or 408 mEq/l). For calcium replacement, we initially used calcium chloride 184 mEq/l (calcium 92 mmol/l) and later calcium chloride 460 mEq/l (Life®) (calcium 230 mmol/l).

The prescription used for dialysis was structured in a very simplified way to facilitate the management of the dialysis session, beginning with the administration of 4% sodium citrate with an infusion flow of 260ml/hour in most patients. This flow results in a final citrate concentration between 2 and 5 mmol/l in the blood, as the prescribed blood flows were between 100 and 250 ml/min. The prescription for additional calcium replacement was with a flow of 8 ml/h (calcium chloride 460 mEq/l) or 20 ml/h (calcium chloride 180 mmol/l). The dialysate calcium was at a final concentration of 1.25 mmol/l. This prescription results in low operational demand on the part of nursing and simple handling for all teams, which has become especially important during the COVID-19 pandemic.

The blood and dialysate flow were mainly the same, and the definition of the flow was based on the attending physician’s decision, considering the clinical condition of the patients (especially regarding the hemodynamic status and dialysis duration). The volume of dialysate prescribed is 90 L, and the time of viable therapy depends on the dialysate flow, although there is the possibility of supplying dialysis to the patient for 24 h with a 2:1 line, which allows a dialysate flow of 60 ml/h with a blood flow of 120 ml/h since this line is 50% of the diameter of the blood flow line.

Patients older than 18 years of both sexes, admitted to the ICU with a diagnosis of AKI, who underwent prolonged HD in a Genius® hybrid system with a 4% sodium citrate solution were included. Exclusion of pregnant women and patients prescribed isolated ultrafiltration from the study. The definition and classification of AKI used is the one recommended by KDIGO [10], defining AKI as urinary volume less than 0.5ml/kg/h for more than 6 h or an increase in serum creatinine > 0.3 mg/dl in 48 h or > 50% in 7 days. AKI was classified into 3 stages (I, II, and III) as recommended by the guideline [10].

Primary outcomes were described as Effectiveness Indicators (minimum completion of 80% of prescribed HD time and system or catheter thrombosis) and Safety Indicators (dysnatremia and metabolic alkalosis). Secondary outcomes evaluated were: prescribed HD time, completed HD time, death from all causes, and pre- and post-session biomarkers (sodium, bicarbonate, calcium, hemoglobin, potassium, urea).

COVID-19 was found to be a potential effect confounder. However, data analysis showed no significant relevance, which will be presented in the results. As this is a retrospective study, the main effort to minimize selection biases was the random inclusion of dialysis sessions that made up the sample. This procedure will be described in detail below.

The present study was carried out with secondary data from electronic medical records, combining two techniques, scripted and manual data collection. Initially, specific scripts were built for massive data collection through reports from the hospital information system. As a result, a primary dataset was constituted, which contained 18,591 HD prescriptions in the Genius® hybrid system performed at the PUCRS HSL from June 12, 2018, to April 13, 2022.

Afterward, minimal data preprocessing occurred, and an experimental dataset was constituted with 3,671 HD sessions in Genius® using citrate as an anticoagulant. The sampling of the sessions occurred through randomization of the experimental dataset using a sample calculation technique based on the optimal allocation criterion for complex sampling with the generation of 100 random sessions of prolonged HD with RCA. It is noteworthy that punctual intermediate sessions were selected, that is, those that occurred between the patient’s first and last session. Subsequently, manual collection of information of interest was performed in the evolution of medical records, the Renal Replacement Therapy (RRT) flow sheet, or both.

The mixed subsampling technique is advantageous when the primary dataset has a significant volume of data. The sample size can interfere with the conclusions about the tested phenomenon, so this technique helps when using information from punctual estimates in big data. Thus, a subsample is defined to use the variability estimates of the estimators, given a previously established error and confidence level.

All statistical analyzes were performed with R Studio® for Windows® version 4.2.2. The application of the Shapiro-Wilk test demonstrated the non-normality of the data. The usage of the Holm-Bonferroni method corrected multiple comparisons and alpha error hyperinflation. P value < 0.05 was considered for statistical significance.

The singular generalized additive model was performed to assess the association between the prescribed time and the completed time of HD in all sessions and in the subgroup with COVID-19, allowing for numerous types of dependent and independent variables. Therefore, it is a semiparametric approach [18].

At the same time, Kendall’s rank correlation coefficient was calculated [19]. The 95% confidence interval (95% CI) was calculated for all measurements through resampling (Bootstrapping) using the percentile technique with 100 simulations.

The graphical representation of times was performed with Local Regression (Loess), a non-parametric method that estimates curves and surfaces through smoothing with a polynomial of the second degree and span or bandwidth parameter of 0.9 [20].

The data collected were stored on the REDCap® platform (Research Electronic Data Capture) to guarantee the confidentiality and secrecy of the information. This study was approved by the University Ethics Committee with the report No. 5,434,821 on May 27, 2022.

## Results

**Fig. 1:**
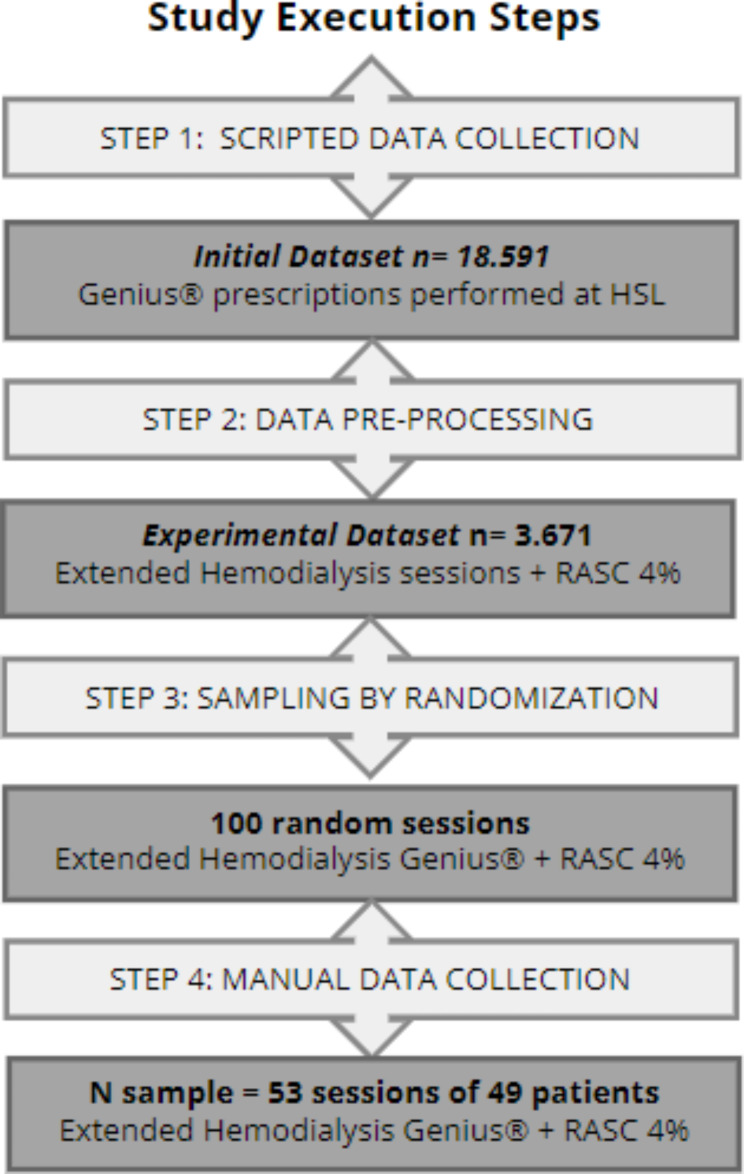
Flowchart for inclusion and exclusion of data and research subjects. RASC, Regional anticoagulation with sodium citrate. Authorship: Isabel Cristina Reinheimer

After the random selection of 100 prolonged HD sessions in Genius® with RCA using a 4% sodium citrate solution, as described above, the manual collection to search for variables of interest began. Those subjects who did not meet the eligibility criteria or had missing data in the electronic medical record and/or flow sheet were excluded. Thus, a sample of 53 random HD sessions of 49 critically ill patients with AKI was constituted (Fig. 1), occurring from December 2019 to December 2021.

Table 1 presents data regarding the characterization of the sample.

**Table 1:** Study sample characterization variables

Variables (n = 49 patients, 53 sessions)	Measurements
Men, *n* (%)	37 (69.8)
Age (years), median (IQR)	53.8 (40-68)
Weight (kg), median (IQR)	79.94 (69-87)
ICU stay days, median (IQR)	30.73 (15-40)
Therapy indication	
Refractory hyperkalemia, *n* (%)	10 (18.9)
Refractory metabolic acidosis, *n* (%)	14 (26.4)
Uremic syndrome, *n* (%)	5 (9.4)
Refractory hypervolemia, *n* (%)	14 (26.4)
Dysnatremia, *n* (%)	6 (11.3)
Intoxication, *n* (%)	5 (9.4)
Anuria, *n* (%)	38 (71.6)
Blood flow (mL/min), median (IQR)	150 (120-150)
Sodium citrate flow 4% (mL/h), median (min-max)	260 (130-270)
Dose of sodium citrate (mmol/h), median (min-max)	30 (15-31)
Calcium gluconate replacement flow (mL/h), median (min-max)	22.0 (8-25)
Dose of calcium replaced (mmol/h), median (min-max)	1.84 (1.84-2.30)
Comorbidities	
CKD, *n* (%)	28 (52.8)
Coronary artery disease, *n* (%)	14 (26.4)
Diabetes Mellitus, *n* (%)	27 (50.9)
Arterial Hypertension, *n* (%)	32 (61.5)
Obesity, *n* (%)	17 (32.1)
COVID-19, *n* (%)	29 (54.7)
General in-hospital mortality, *n* (%)	45 (84.9)
In-hospital mortality from COVID-19, *n* (%)	27 (93.1)
CKD, *n* (%)	14 (26.4)
Stage GFR 2, *n* (%)	1 (1.9)
Stage GFR 3, *n* (%)	2 (3.8)
Stage GFR 4, *n* (%)	5 (9.4)
Stage GFR 5, *n* (%)	6 (11.3)
AKI on arrival from the ICU, *n* (%)	45 (84.9)
KDIGO I, *n* (%)	4 (9.3)
KDIGO II, *n* (%)	10 (23.3)
KDIGO III, *n* (%)	29 (67.4)
Administration of vasoactive drug, *n* (%)	34 (64.2)
Mechanical ventilation, *n* (%)	43 (82.7)

IQR, interquartile range; ICU, intensive care unit; CKD, chronic kidney disease; GFR, glomerular filtration rate; KDIGO, Kidney Disease Improving Global Outcomes (AKI Staging)

Note: Data were expressed as median and IQR for quantitative variables. Minimum and maximum values were used for the sodium citrate and calcium gluconate flow results

Table 1 shows the analysis of the parameters of the dialysis sessions. Table 2 presents the patient’s sodium, bicarbonate, calcium, urea, potassium, and hemoglobin parameters, and the effectiveness indicators evaluated in this study are shown in Table 3.

**Table 2:** Results of Study Safety Indicators and Study Biomarkers

Safety Indicators		
Variables (n = 53 sessions)	Pre session	Post session
HCO_3_ (mmol/L), median (IQR)	25.5 (22.3-28.2)	25.85 (23.8-28.25)
Na, (mEq/L), median (IQR)	138 (135-141)	136 (134-140)
Metabolic Alkalosis, *n* (%)		5 (9.4)
Dysnatremia, *n* (%)		8 (15.0)
Hypernatremia, *n* (%)		1 (1.8)
**Biomarkers**		
**Variables** (n = 53 sessions)	**Pre session**	**Post session**
Ur (mg/dL), median (IQR)	107 (59-166)	114 (66-162)
iCa* (mg/dL), median (IQR)	4.39 (4.1-4.71)	4.56 (4.04-4.9)
K (mEq/L), median (IQR)	4.3 (3.6-4.8)	4.3 (3.6-4.9)
Hb (g/dL), median (IQR)	7.9 (6.8-9.2)	8.05 (6.9-8.7)

* Data observed in 15 of the 53 sessions

Cr, creatinine; Ur, urea; Na, sodium; HCO3, bicarbonate; iCa, ionic calcium; K, potassium; Hb, hemoglobin; IQR, interquartile range. Note: Sample number = 49 patients, 53 sessions

**Table 3:** Results of the effectiveness indicators evaluated in the study

Variables (n = 53 sessions)	Measurements
Pause of Therapy, *n* (%)	12 (22.6)
Reason for the pause	
Hemodynamic instability, *n* (%)	2 (3.7)*
Catheter thrombosis, *n* (%)	1 (1.9)
System coagulation, *n* (%)	9 (17.3)
Prescribed time (hours), median (IQR)	12.32 (8-15)
Run time (hours), median (IQR)	10.5 (6-12)
Total of sessions, *n*	53
Completeness of prescribed time	
100-80%, *n* (%)	40 (75.5)
79-70%, *n* (%)	1 (1.89)
69-60%, *n* (%)	2 (3.77)
< 60%, *n* (%)	10 (18.9)
COVID-19 patients, *n*	29
Completeness of prescribed time	
100-80%, *n* (%)	22 (75.9)
79-70%, *n* (%)	1 (3.4)
69-60%, *n* (%)	1 (3.4)
< 60%, *n* (%)	5 (17.2)
COVID-19 patients with VAD, *n*	20
Completeness of prescribed time	
100-80%, *n* (%)	15 (75)
79-70%, *n* (%)	1 (5)
69-60%, *n*	0
< 60%, *n* (%)	4 (20)

*Pause in the session occurred in 2 patients with COVID-19 who presented hemodynamic instability

VAD,vasoactive drug; IQR, interquartile range

The median prescribed dialysis time was 12.32 h, as shown in Table 3. It is noteworthy that a 24-hour period was prescribed in 6 sessions, having 100% execution completeness in 4 sessions, one achieved 12 h and the other only 4 h.

Pause in therapy occurred in 22.6% of the sessions, of which: 3.7% occurred due to hemodynamic instability, 1.9% due to catheter thrombosis, and 17.3% due to system coagulation. When observing completeness ≥ 80% of the prescribed time in patients with COVID-19, the percentage was 75.9% concerning 75.5% of all sessions evaluated. Thus, regarding the non-parametric regression criterion, there is no difference between the prescribed and performed time for patients affected by COVID-19, presenting the following regression equation: 1.76 (95% CI: -1.95; 5.47, P = 0.36) + 0.70*prescribed time (95%CI: 0.45; 0.97, P < 0.001) − 1.77*COVID-19 (95%CI: -6.67; 3.11, P = 0.48) + COVID-19/prescribed time*0.18 (95%CI:: -0.18; 0.54, P = 0.33).

## Discussion

The present study described, for the first time, in a Brazilian university hospital, the results of effectiveness and safety indicators in RCA with a 4% sodium citrate solution in prolonged HD with the Hybrid Genius® System in critically ill patients with AKI.

### Profile of patients and clinical outcomes

In the present study, the outcomes of length of stay (30.7 days), general in-hospital mortality (84.9%), and in-hospital mortality in COVID-19 (93.1%) were high. The proportion of patients who achieved KDIGO stage 3 AKI was 67.4%. A prospective cohort carried out at a public university hospital in São Paulo in the first 90 days of the pandemic evaluated 101 patients hospitalized with COVID-19, 51.9% of which required an ICU. Of these critically ill patients, 77.3% developed AKI, and the third KDIGO stage was the most frequent (58.9%). Acute RRT was indicated in 61.5% of patients, and mortality was 65.4% [21].

In the present study, most patients (84.9%) had AKI on admission to the ICU, denoting a probable more severe clinical picture of the studied sample. This sample consisted of 29 patients with COVID-19, representing 59.18% of the total. Of these, 68.96% were receiving vasoactive medications, and 82.7% were on mechanical ventilation. Therefore, a higher occurrence of mortality is expected.

### Pre- and post-dialysis biomarkers

Ionic calcium increased slightly post-session (4.39–4.56 mEq/l) but remained in the normal range (4.5–5.3 mg/dl). The dialysis solution available at the service contains calcium, but calcium chloride infusion probably influenced these values. Calcium monitoring when using RCA is justified because this medication reduces iCa in the extracorporeal circuit (since it is an essential cofactor of the coagulation cascade). Up to 50% of the citrate-calcium complex is removed through the hemofilter during the first pass. Then, calcium gluconate infusion may be necessary to avoid a negative balance and compensate for calcium losses. However, not all protocols recommend this infusion [13].

Urea was above the reference value (19–43 mg/dl) in the pre-session and urea increased in post-session concentration (107–114 mg/dl). Among the possible limitations that justify it is the time of test collection, which did not occur shortly after the end of dialysis (recalling that it occurred during the COVID-19 pandemic). This limitation in this retrospective study is due to the fact that there is no research protocol. In this regard, it is also known that urea is a marker of nutritional performance. HD patients have a high prevalence of malnutrition [22]. In general, critically ill patients may have 20% muscle loss in the first 10 days of hospitalization [23].

Is important to note that the Hybrid Genius® System is a unique hemodialysis system that has the ability to keep the fresh and waste dialysate separated, despite both being stored on a unique reservoir. This is possible because of the dialysate tank, a glass container with a capacity to store 90 L of dialysis solution. Both dialysates, the used and the fresh, are kept in the same tank, but they do not mix. The difference in density and temperature is what keeps both liquids separated, were the wasted dialysate will remain on the bottom of the tank with the fresh one above it [6].

### Safety and effectiveness indicators

The main disadvantage of RCA use is metabolic and electrolyte disturbances (hypocalcemia, metabolic alkalosis, and hypernatremia) [24]. As shown in Table 2, the analysis of safety indicators showed that post-session sodium and bicarbonate concentrations remained within reference values. Dysnatremia occurred in 15% of sessions, and metabolic alkalosis was observed in 9.4% of those.

In the present study, the median flow of 4% sodium citrate infused in patients was 260 ml/h, with a citrate dose of 30 mmol/h. The median flow of the calcium chloride replacement solution was 22 ml/hour, with a calcium dose of 1.84 mmol/h. The occurrence of hypernatremia was evidenced in 1 session (1.8%). In the retrospective study by Wen et al., which evaluated 808 sessions with RCA with a 30% citrate solution, 1 HD session was interrupted due to sodium disturbance [11]. In this regard, the literature describes hypernatremia as a potential adverse event of RCA, with an uncommon occurrence when using a dialysate or replacement fluid with lower sodium concentrations or both [25, 26]. The infusion of concentrated sodium citrate resulted in sodium overload for the patient (420 mmol/l in a 4% solution and 3,060 mmol/l in a 30%), but this was not a significant problem in our study. Even using a dialysate with 135 mmol/l of sodium, dysnatremia was uncommon, hypernatremia being a rare event. In addition, the sodium content of other infusions and intravenous fluids, in addition to enteral feeding, must be considered to maintain the patient’s electrolyte balance [12].

Another potential adverse event in RCA is metabolic alkalosis. As previously described, the filter removes up to 50% of the citrate-calcium complexes. Those that enter the systemic circulation dissociate, and the Krebs cycle metabolizes citrate (mainly in liver cells). In the end, one citrate molecule produces energy and three bicarbonate molecules. Therefore, sodium citrate acts as both an anticoagulant and a buffer base. Thus, the acid-base balance of patients should be monitored since there is a risk of metabolic alkalosis [13, 14].

In the study by Wen et al., four interruptions of the dialysis session due to electrolyte and metabolic disturbances were observed, two of which were metabolic acidosis. This study reported no interruption due to alkalosis [11]. In the present study, the bicarbonate concentration increased after the session (25.5–25.85 mmol/l), remaining within the reference values ​​(22–28 mmol/l). However, metabolic alkalosis was observed in 5 sessions, and there was no detection of acidosis occurrence.

Regarding the effectiveness indicators shown in Table 3, system coagulation occurred in 17.3% of the HD sessions. A similar result was described by Schneider et al., which showed 19% coagulation with RCA using a 4% sodium citrate solution [25]. Wen et al. reported circuit coagulation in 38 sessions (5% of the total), yet, the RCA concentration was 30%. [11].

The second effectiveness indicator evaluated in the present study was the minimum completeness ≥ 80% of the prescribed HD time, which was 75.5% (Table 3). When assessing the sessions that reached 100% of the prescribed HD time, the result was 69.8%. A similar percentage appeared in the prospective study by Schneider et al., in which full dialysis time occurred in 73 of the 103 treatments (71% of sessions). However, the study occurred with 34 patients divided into 6 anticoagulation protocols. Only 11 sessions were performed with RCA using a 4% sodium citrate solution [25].

### Quality indicators (QIs) in acute dialysis

In ICUs and dialysis units, it is essential to measure dialysis performance to promote treatment effectiveness and patient safety [27]. For this, information supported by valid and reliable data is imperative when analyzing the treatment provided [28] and developing quality indicators [29].

Quality Indicators (QIs) represent methods for evaluating the performance of target areas of the health system. They seek to measure, monitor, analyze, and communicate the effectiveness of actions and services. Generally, QIs use the Donabedian triad, which proposes the evaluation of health care through Structure Elements (where they are provided), Process Elements (how they are provided), and Outcome Elements (the effects of care delivery). In the present study, QIs that are similar to the parameters of two domains of the referred method were analyzed: Process Element (minimum completeness of prescribed HD time) and Result Elements (dysnatremia and metabolic alkalosis and system coagulation).

One study sought to develop a method to assess how improving the quality of dialysis delivered to patients could impact clinical outcomes. Three categories of objective metrics were established for the filter, prescription, and fluid balance in order to perform quarterly reviews to drive staff training and measure care performance. A total of 184 critically ill patients on continuous HD from 2012 to 2017 were evaluated. The study concluded that implementing QIs can support the development of metrics to assess the performance of institutional standards of continuous HD, specifically in compliance with the proposed care [30].

Again, it is worth mentioning that no specific studies that addressed Quality Indicators in prolonged HD were found. Nor were they carried out in a hybrid system using RCA at a 4% concentration. Thus, the present study proposed to present data that could contribute to the discussion on the subject and provide insights that collaborate with new research in the area.

## Limitations

The present study did not evaluate direct safety measures (which would be the accumulation of sodium citrate in the serum) and the effectiveness of anticoagulation (control of the iCA level in the circuit, which is performed through a port located after the hemofilter). The present study evaluated indirect measures (which were the biomarkers and aspects related to the dialysis session). Based on this, it is essential to carry out prospective studies with research protocols based on clinical practices that produce results that can guide care therapy and work processes.

## Conclusion

The Quality Indicators evaluation showed that using RCA with a 4% sodium citrate solution in prolonged HD in critically ill patients with AKI can be performed safely, effectively, and in a simple way using the Genius® hybrid system. Thus, hypertonic sodium citrate use is an alternative for anticoagulation in this dialysis system. In conclusion, the QIs results are relevant parameters for evaluating acute RRT delivered to patients.

## Data Availability

The datasets used and/or analysed during the current study available from the corresponding author on reasonable request.

## Abbreviations

HD: Hemodialysis
RCA: Regional Citrate Anticoagulation
IQs: Quality Indicators
AKI: Acute Kidney Injury
ICU: Intensive Care Units
HTA: Health Technology Assessment
ADQI: Acute Disease Quality Iniciative
HSL: Hospital São Lucas
PUCRS: Pontifical Catholic University of Rio Grande do Sul (PUCRS)
RRT: Renal Replacement Therapy
